# Key Factors in the Quality of Life of African American Parents Living With Dementia

**DOI:** 10.1093/geroni/igaf122.2922

**Published:** 2025-12-31

**Authors:** Daum Chung, Kalisha Bonds Johnson

**Affiliations:** Emory University, Atlanta, Georgia, United States; Emory University, Atlanta, Georgia, United States

## Abstract

Relationship quality is crucial for the quality of life (QoL) of people living with dementia (PLWD), yet cognitive impairment often limits their participation in decision making. This cross-sectional secondary data analysis aimed to investigate how QoL of African American parents was influenced considering life course. Data were originally collected from 39 African American parent–adult daughter dyads. Parent data with no missing values (N = 33) were included in the analysis. PLWD reported their age, QoL, self-rated health, their perceptions of the dyadic relationship quality, and their perceived level of difficulty in having their opinions heard by family members. PLWD’s dementia severity was assessed by their adult daughters using the Dementia Severity Rating Scale. In the final hierarchical regression model, “good” and “very good” self-rated health, positive interaction of the dyadic relationship, and having “some” difficulty in having opinions heard by family members were identified as significant predictors of QoL. Better self-rated health and positive interaction in the relationship quality were associated with better QoL, while greater difficulty in having their opinions acknowledged by family was associated with worse QoL. Findings suggest that maintaining a positive dyadic relationship alone is not sufficient to enhance African American PLWDs’ QoL. Their perceptions of how difficult it is to have their opinions heard by their family members seem to matter. Therefore, QoL intervention for African American PLWD may benefit from strengthening positive interaction in the dyadic relationship while fostering strategies to support parents’ opinions are heard without difficulty.

